# Multibeam inverse intensity-modulated radiotherapy (IMRT) for whole breast irradiation: a single center experience in China

**DOI:** 10.18632/oncotarget.5278

**Published:** 2015-09-16

**Authors:** Zhaozhi Yang, Li Zhang, Xingxing Chen, Jinli Ma, Xin Mei, Jiayi Chen, Xiaoli Yu, Xiaomao Guo

**Affiliations:** ^1^ Department of Radiation Oncology, Fudan University Shanghai Cancer Center, Department of Oncology, Shanghai Medical College, Fudan University, Shanghai, China; ^2^ Department of Radiation Oncology, Ruijin Hospital, Shanghai Jiaotong University School of Medicine, Shanghai, China

**Keywords:** breast cancer, radiotherapy, IMRT, whole breast irradiation, breast conservative treatment

## Abstract

**Purpose:**

To present the clinical experience in our cancer center with multibeam inverse intensity-modulated radiotherapy (IMRT) for early stage breast cancer (BC) patients with whole breast irradiation (WBI).

**Methods:**

We retrospectively analyzed 622 patients with Stage 0 to III BC treated from 2008 to 2011 with wide local excision and WBI, using an inverse IMRT technique. All of the patients were prescribed a total dose of 50 Gy to the whole breast in 2-Gy fractions, followed by a tumor bed boost of 10 Gy in 5 fractions using an electron beam.

**Results:**

Of all of the patients, 132 (21.2%) received whole breast plus regional lymph node (RLN) irradiation. 438 of 622 patients had records of acute skin toxicity based on common terminology criteria (CTC) for adverse events. Two hundred eighty (64%) patients had Grade 0/1 toxicity, 153 (35%) had Grade 2 and only 4 patients experienced grade 3 toxicity. Seventy patients (16%) had moist desquamation. Univariate analysis revealed that breast planning target volume was the only predictive factor for Grade ≥2 acute dermatitis (*P* = 0.002). After 4 years, 170 patients reported cosmetic results by self-assessment, of whom 151 (89%) patients reported good/excellent cosmetic results, and 17 (11%) patients reported fair assessments. For invasive cancer, the four-year rate of freedom from locoregional recurrence survival was 98.3%. Regarding carcinoma *in situ*, no patients experienced recurrence.

**Conclusion:**

BC patients who underwent conservative surgery followed by inverse IMRT plan exhibited acceptable acute toxicities and clinical outcomes. Longer follow-up is needed.

## INTRODUCTION

Breast conservative treatment exhibits disease-free survival and overall survival rates that are similar to those for mastectomy in early stage breast cancer (BC) patients [1, 2]. A systemic review identified that postoperative radiotherapy (RT) not only reduced locoregional recurrence, but also improved the BC-specific survival [3]. Whole breast irradiation (WBI) is typically delivered by two opposing tangential fields directed to the breast at an angle approximately parallel to the chest wall. RT-related complications, such as acute dermatitis, breast fibrosis and telangiectasis, have influenced cosmetic results and the quality of life of patients [3–7]. Moreover, non-breast related mortality and morbidity from late cardiovascular damage as a result of cardiac exposure to radiation is counterbalanced in part by the survival gain [8]. The potential for cardiac toxicity has recently increased given the more widespread use of anthracyclines/taxanes/trastuzumab in the adjuvant treatment of early-stage BC [9].

Randomized trials have demonstrated that forward IMRT reduces moist desquamation and late breast appearance changes, improving cosmetic result [4, 6, 7]. However, numerous studies have also confirmed improved cardiac dosimetry with inverse IMRT by reducing the volume of high-dose irradiation of the heart when treating the whole breast and/or regional lymph nodes (RLNs) compared with three-dimensional conformal radiation therapy (3DCRT) [10–16], particularly in patients with unfavorable chest-heart geometry or those receiving RLN irradiation involved.

Several studies have demonstrated that the volume of the heart receiving high dose was substantially reduced in IMRT plans with nine equispaced fields [11, 13, 17]. Considering the widespread low-dose irradiation of the normal tissue and its unknown potential risks for patients, Multibeam IMRT with tangential orientation is believed to offer optimum balance of target coverage and normal tissue sparing with treatment complexity [10, 18–20]. However, because of the higher costs of IMRT, Only 10% patients received inverse IMRT for BC in the United States in 2011 [21]. IMRT for accelerated partial breast irradiation (APBI) recently achieved good clinical result in Italian single center study [22]. In addition, the IMRT-MC2 study which is a two-armed, multicenter, randomized, phase III trial compared WBI with simultaneously integrated boost by inverse IMRT technique with sequential boost after WBI [23]. But the clinical experience of inverse IMRT for WBI with conventional fraction has only rarely been reported.

We introduced the WBI with multibeam IMRT technique with 4 to 7 gantry angles since in April 2008. In this study, we will present the clinical experience of our cancer center.

## RESULTS

### Patient and treatment characteristics

The characteristics of the 622 patients are presented in Table 1. Of all of the patients, 92 patients (14.8%) had carcinoma *in situ*, 367 patients (59%) had T1 disease and 146 patients (23.5%) had T2 disease. In total, 24% of the patients had positive lymph nodes. Twelve patients had close margins. Of all of the patients, 78% patients underwent endocrine therapy and 75% received chemotherapy. Of the patients with invasive cancer, 51patients received trastuzumab treatment for one year. One hundred thirty-two (21.2%) patients received whole breast plus RLN irradiation, 8 had internal mammary node irradiation and 35 patients were treated with a separately matched supraclavicular field.

**Table 1:** Patient characteristics

Characteristics	All Patients (*n* = 622)	Invasive cancer (*n* = 530)	Carcinoma *in situ* (*n* = 92)
Age at diagnosis			
Median(range)	45(21–78)	45.5(21–78)	45(25–64)
Primary histology (n)			
Carcinoma *in situ*			92
IDC		494 (93.2)	
Others histology		36 (6.8)	
Breast side			
Left	304 (48.9)	269 (50.7)	35 (38)
Right	318 (51.1)	261 (49.3)	57 (62)
T stage			
Tis	92 (14.8)		92 (100)
T1	367 (59)	367 (69.2)	
T2	146 (23.5)	146 (27.5)	
unknown	17 (2.7)	17 (3.2)	
Sentinel node sampled (n)			
Median (range)		3 (1–8)	3 (1–6)
Lymph node clearance (n)			
Median (range)		16 (2–41)	0 (0–23)
N stage			
N0	435 (69.9)	383 (72.2)	52 (56.5)
N+	147 (23.6)	147 (27.7)	
Nx	40 (6.4)		40 (43.5)
ER status			
Positive	454 (73.0)	385 (72.6)	69 (75)
Negative	162 (26.0)	142 (26.8)	20 (21.7)
Unkown	6 (1.0)	3 (0.5)	3 (3.3)
PR status			
Positive	433 (69.6)	368 (69.4)	65 (70.6)
Negative	184 (29.6)	160 (30.2)	24 (26.1)
unkown	5 (0.5)	2 (0.4)	3 (3.3)
HER2 status			
Positive		93 (17.5)	7 (9.6)
Negative		434 (81.8)	82 (89.1)
unknown		5 (0.7)	3 (3.3)
Molecular subtype			
Luminal		400 (75.5)	
Triple negative		90 (17.0)	
HER2 enriched		38 (7.1)	
Unknown		2 (0.4)	
Grade			
I-II	398	317 (59.8)	72 (78.2)
III	161	147 (27.7)	12 (13.0)
unkown	75	66 (12.5)	8 (8.7)
LVI			
Positive		81 (15.3)	
Negative		344 (64.9)	
unkown		105 (19.8)	
Margin			
Positive/close	12 (1.9%)	11 (2.1)	1 (1)
Negative	582 (93.5)	499 (94.2)	83 (90.2)
Unkown	28 (4.5%)	20 (3.8)	8 (8.7)
Endocrine therapy	482	406	76
Chemotherapy	470	446	24
Trastuzumab	51	51	0
Radiotherapy			
Breast only	490 (78.8)	398	92
Breast + RLN	132 (21.2)	132	0

*Abbreviation*: Nx lymph node not be evaluated; IDC invasive ductal carcinoma; LVI lymphovascular invasion

### Treatment plan parameters

Of all of the patients, 438 patients had detailed depictions of acute skin toxicity during and after irradiation. Dose volume histograms of these 438 patients were evaluated. The mean planning target volume (PTV) was 520 cc and the average mean heart dose (MHD) was 610 cGy for left breast irradiation. The mean percentage volume of heart receiving ≥30 Gy (V30) of al l of the left-sided breast patients was 3.2%. The treatment plan parameters for these patients are presented in Table 2.

**Table 2:** Selected dosimetric parameters for 438 patients

Parameters	Mean	SD	Range
Breast PTV (cc)	522	212	165–1149
PTV_95%_ (%)	97.5	1.1	94.3–99.1
PTV_107%_(%)	3.3	4.8	0–18.2
Ipsilateral Lung V_20_ (%) (breast only)	19.0	2.9	13.0–27.1
Ipsilateral Lung V_20_ (%) (breast and RLN)	25.0	5.3	16.8–35.4
Mean heart dose (cGy) (left breast)	610	260	158–1147
V_30_ of heart (%) (left breast)	3.2	1.9	0–9.5

Abbreviation: SD standard error

### Acute skin toxicity and cosmetic result

Two hundred eighty (64%) patients had Grade 0/1 acute skin toxicity, 153 (35%) patients had Grade 2 toxicity and only 4 patients had Grade 3 toxicity. Seventy patients (16%) had varying extents of moist desquamation in the axilla, inframammary and/or nipple areola. During RT, 3 patients (0.7%) developed cellulitis and were treated with antibiotics. Univariate analysis revealed that age, the percentage volume of PTV receiving ≥107% prescribed dose (PTV_107%_), RLN irradiation and chemotherapy had no significant value in predicting the occurrence of Grade ≥2 acute skin toxicity. However, breast PTV was the only predictive factor (*p* = 0.002) (Table 3). Small breast PTVs (≤520 cc) resulted in 26% of cases developing Grade 2 or higher skin toxicity, whereas larger PTVs (>520 cc) resulted in this complication in 41% of patients. After 4 years, the 170 patients who agreed to assess their cosmetic results judged their cosmetic results by self-assessment. Of these patients, 151 (89%) reported good/excellent cosmetic results, whereas 19 (11%) patients reported fair assessments.

**Table 3:** Univariate analysis for factors affecting the acute dermatitis

Variables	Odds Ratio (95% CI)	*P* value (≥grade 2 acute skin toxicities)
Age	0.889 (0.606,1.304)	0.547
PTV (≤520 vs. > 520 cc)	0.540 (0.363, 0.801)	0.002
Breast PTV_107%_ (≤3.3% VS. > 3.3%)	0.687 (0.464,1.107)	0.071
Chemotherapy (yes vs. no)	1.105 (0.624, 1.416)	0.766
RLN (yes vs. no)	1.025 (0.682,1.540)	0.906

### Clinical outcomes

Of the 530 patients with invasive BC, the median follow-up duration was 51 months (range, 4–76 months), and 53% of the patients experienced greater than 4 years of follow-up. The events after the completion of treatment are shown in Table 4. Five patients had in-breast recurrence, 3 patients had local recurrence alone and 2 patients had simultaneously axillary recurrence and/or distant metastasis. Eight patients had axillary recurrence, but only one had isolated axillary recurrence. Twenty-three patients had distant metastasis. The four-year rates of LRRFS, DMFS, RFS and OS were 98.3%, 96.1%, 94.9% and 97.9%, respectively. The univariate and multivariate analyses of potential risk factors for recurrence are shown in Table 5. Only T2 stage (hazard ratio [HR], 2.01; 95% confidence interval (CI), 1.04–4.79; *P* = 0.013), triple negative subtype (HR, 3.01; 95%CI, 1.34–7.12; *P* = 0.001) and HER2-enriched subtype (HR, 2.15; 95%CI, 1.21–5.53; *P* = 0.008) remained statistically significant in multivariate analyses.

**Table 4:** Events during the follow-up for invasive cancer

Event	Number	Incidence (%)
Locoregional recurrence	11	2.1%
In-breast recurrence	5	
Axillary node	8	
Supraclavicular node	1	
Internal lymph node	1	
Distant metastasis	23	4.3%
Contralateral breast cancer	5	0.9%
Non-breast Secondary cancer	7	1.3%
Thyroid cancer	3	
cervical cancer	2	
Sarcoma (leg)	1	
Urothelial cancer	1	
Death	11	2.1%

**Table 5:** Univariate and multivariate analysis of recurrence free survival for 530 invasive breast cancer patients

	Univariate		Multivariate	
Factors	HR(95% CI)	*P* value	HR (95% CI)	*P* value
Age	0.96 (0.86–1.12)	0.173		
T (T2 vs. T1)	2.23 (1.02–4.89)	0.007	2.01 (1.04–4.79)	0.013
N (N+ vs.N0)	1.34 (0.89–3.45)	0.254		
LVI(yes vs. no)	1.43 (0.67–3.87)	0.336		
Grade (III vs. I+II)	1.88 (0.90–4.02)	0.052		
Molecular subtype				
TN vs. Luminal	2.69 (1.18–6.16)	0.001	3.01 (1.34–7.12)	0.001
HER2 vs. Luminal	2.01(1.62–7.34)	0.012	2.15 (1.21–5.53)	0.008

Abbreviation: HR hazard ratio

Of the 92 patients with carcinoma *in situ*, the median follow-up duration was 49 months (range, 16–69 months). No patients experienced recurrence. However, 2 patients had thyroid cancer during the follow-up, and 100% patients survived until the last follow-up. Of the 622 patients, 14 (2.2%) developed the secondary primary cancers which included 5 thyroid cancers, one cervical cancer, one urothelial cancer and one sarcoma after treatment.

## DISCUSSION

Our study demonstrated that inverse multibeam IMRT for WBI exhibited an acceptable acute skin toxicity profile and cosmetic results by patient's self-assessment. After 4 years of follow up, the locoregional recurrence was low.

In clinical practice, randomized and non-randomized data have demonstrated the superiority of RT when delivered with IMRT compared with standard tangential RT. Vicini et al. [24] firstly reported the toxicity profile of forward IMRT. In that study, 56% of patients developed Radiation Therapy Oncology Group (RTOG) Grade 0 to 1 acute skin toxicity, 43% developed Grade 2 acute skin toxicity and 1% experienced Grade 3 toxicity. In addition, the target received 105% and 110% of the prescribed dose thus significantly predicting skin toxicities. McDonald et al. [25] compared conventional RT with forward IMRT and found that acute skin toxicity of RTOG Grade 2 or 3 was reduced from 52% to 39%. A Canadian randomized study [6] and another retrospective study [26] showed that forward IMRT reduced moist desquamation, which was evaluated using CTC for toxicity. The moist desquamation rates decreased from 38% and 47.8% in patients with standard RT to 21 and 31.2% in patients with IMRT. In these two studies, small breast size was significantly associated with decreased moist desquamation.

However, clinical experience with inverse IMRT planning has only been reported in patients with WBI with simultaneously integrated boosts. McDonald et al. [27] reported 354 Stage 0 to III BC patients underwent conservative surgery and postoperative RT. In this study, 43% of the patients experienced CTC Grade 2 acute toxicity. Another hypofractionated RT study also used inverse IMRT plan to treat patients in the prone position and the skin toxicities with RTOG Grade ≥2 were only 13%. Our data demonstrated that WBI with 50Gy in 25 fractions and inverse IMRT planning exhibited a similar toxicity profile with previous study. We found breast PTV is the only predicting factor for ≥Grade 2 toxicity. However, our moist desquamation rate looks lower than previous reports. This finding could be explained by two reasons. Firstly, our mean breast PTV (520 cc) is smaller than that of Western women (≥700 cc) [27, 28] because the breast size of Chinese women are smaller than Western women. Secondly, multibeam IMRT could offer a significantly reduced surface dose compared to forward IMRT with tangential field [29].

Our average MHD for left breast irradiation was similar to the rate that Paul McGale et al. [30] reported. In that investigation of more than 35000 women, the MHD was 6.3 Gy for all left-sided patients from 1980 to 2001. Recent studies have demonstrated that the MHD varied in different studies from 2.6 Gy to 9.0 Gy [18, 27, 31]. Due to interobserver and interinstitutional variability in delineating target volumes, it is difficult to compare MHDs [32, 33]. However, Our V_30_ value was low and acceptable similar to previous dosimetric studies [11, 18]. Clinical experience with Hodgkin disease has demonstrated that a heart dose >30 Gy is associated with an increased rate of cardiac mortality [34, 35], thus reducing the V_30_ could be particularly important. To our knowledge, this report was the first on MHD and V30 in the IMRT era in a large sample size.

Our study had achieved excellent local control rate, the 4 year rate of ipsilateral breast tumor recurrence (IBTR) was 0.9%, which seems to be lower than previous reports. Yau et al. [36] reported that the 5 year IBTR rate was 4% in 412 patients in Hong Kong. Bartelink et al. [37]reported the 5 year local recurrence rate was 7.3% in the boost arm of the EORTC study 22881–10882. However, the enrollment period of patients in these studies was older than that of our patients. So, this could partially be attributed to the modern comprehensive systemic treatment.

One concern with multibeam IMRT is an increased risk of secondary malignancies [38]. Given the multi-directional field arrangements for the target and increased monitor units for delivery, low-dose spread and possibly more scattering of the dose deserve our continued careful consideration and require long-term follow-up. In this study, the 4-year occurrence rates of contralateral BC and secondary malignancy were 0.9% and 2.2%, respectively, for all 622 of the patients. Livi et al. [22] reported the 5-years results of IMRT for APBI. In this study, the 5-year occurrence rate of contralateral BC in the IMRT-APBI group was 1.6%, which is similar to that the standard RT group (3.2%). However, a recent study [39] in which patients were treated with WBI and concurrent integrated boost using a 3DCRT technique also reported 5-year rates of secondary malignancy and contralateral BC of 6% and 2.6%,respectively, in 752 patients. Therefore, whether multibeam IMRT for BC radiation could increase secondary malignancies cannot thus far be concluded. To do so would require a larger sample size study and longer follow-up.

A limitation of this paper was that not all of the patients had acute toxicity records and the follow-up was relatively short. We only reported cosmetic results using patient's self-assessment and without physician assessments or photographic evaluations. We did not report on late skin fibrosis or telangiectasis.

## MATERIALS AND METHODS

### Patient selection and evaluation

Between April 2008 and April 2011, the charts of patients with Stage 0 to III BC who underwent conservative surgery and WBI in the Department of Radiation Oncology at our cancer center were reviewed. Patients with the following conditions were excluded from this retrospective analysis: treatment with preoperative systemic therapy, prior malignancies (except for nonmelanoma skin cancers), and synchronous bilateral BC. This investigation was approved by the Institutional Review Board of Fudan University Shanghai Cancer Center. During this period, the multibeam IMRT technique was initialized by one radiation oncologist in some patients and by all of the physicians in all patients in 2010. Only patients who underwent multibeam IMRT were included in this analysis.

### Surgery

The breast surgery involved wide local excision and level I/II axillary lymph node dissection (ALND) or sentinel lymph node (SLN) biopsy. The majority of patients with positive SLNs, received ALND. Patients with close or positive margins underwent re-excision or secondary mastectomy. Margins were defined as “positive” when the tumor (invasive or carcinoma *in situ*) was observed at the edge of the resection, “close” when the tumor was at a distance of 2 mm or less from the resection edge, and “negative” when this distance was greater than 2 mm [40].

### Systemic treatment

Most of the patients received adjuvant systemic chemotherapy according to St. Gallen and/or National Comprehensive Cancer Network (NCCN) guidelines. Hormone therapy was prescribed to hormone receptor-positive BC patients using tamoxifen or aromatase inhibitors with or without goserelin. Some patients with HER2 overexpression received adjuvant trastuzumab based on their economic status.

### Radiotherapy

Planning CT images were acquired prior to radiation. Each patient was positioned supine on an inclined breast board (MED-TEC, INC. MED-TEC MT-350) with both arms abducted and raised above her head to grip a crossbar. The CT images were obtained at a 5-mm slice thickness using a simulated CT scanner (Philips Medical Systems, Cleveland, OH, USA). The images ranged from the mandible to the mid-abdomen. Before scanning, radiopaque catheters and markers were placed to locate both palpable breasts and scarring and to facilitate breast target volume delineation

**Figure 1:**
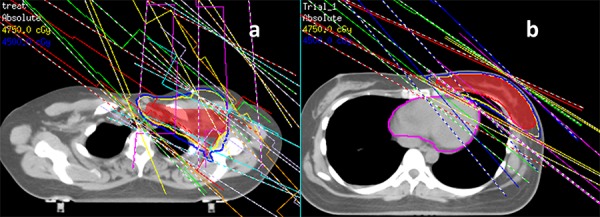
The gantry angles for multibeam inverse IMRT **a.** 7 fields for left breast and regional lymph node (0°,118°,132°,145°, 302°,312°,345°); **b.** 5 fields for left breast alone (300°,310°,325°, 120°,135°).

A step-and-shoot IMRT plan was developed using an inverse planning technique. Initially, the IMRT was exclusively applied to the whole breast, when necessary, the supraclavicular field was added with mixed X-ray and electron anterior fields, which matched the breast plan. However, several months later, the technique was optimized and improved, and the breast and RLN were integrated into one target and optimized. Using the Pinnacle treatment planning (Philips Co.), an IMRT plan with 4 to 7 fields (median of 5 fields) referring to the tangential directions was created by avoiding contralateral breast and lung irradiation (Figure 1). The beam angles were 115 to 145° for medial fields and 300 to 345° for lateral fields in left-sided patients as well as 245 to 220° for medial fields and 60 to 30° for lateral fields in right-sided patients. A perpendicular field (0°) was exclusively used in patients undergoing RLN irradiation. The total dose of 50 Gy in 25 fractions over 5 weeks was prescribed to the whole breast. The dose constraints for optimization were as follows: the percentage volume of the PTV receiving ≥ 95% of the prescription dose (V_95%_) was≥ 95%; the percentage volume of the ipsilateral lung receiving ≥ 20 Gy (V_20_) was ≤20% for patients undergoing breast irradiation alone and 30% for patients undergoing breast and RLN irradiation; The MHD was ≤6 Gy for left-sided patients and a 20% variation in dose limitation for organ at risk (OAR) was permitted. Every attempt was made to make the cardiac dose as low as possible. Once the PTV and OAR dose met the pre-set constraints, an around 2 cm flash beyond the patient surface was added to account for potential setup errors. After WBI, a sequential electron boost of 10 Gy in 5 fractions was added tothe tumor bed and the boost field was perpendicular to the tumor bed with a 1- to 2-cm margin from each clip in the coronal direction.

### Target volume definition

The whole breast clinical target volume (CTV) included the glandular breast tissue of the ipsilateral breast and did not extend into the pectoralis major or the ribs. The PTV was defined by adding a margin of 5 mm in the anterior-posterior direction and 10 mm in the medial-lateral and superior-inferior directions. The PTV was limited superficially to 5 mm beneath the skin surface. Supraclavicular and infraclavicular CTVs were contoured according to published CT-based nodal references [41]. The IM node was represented with a contour of the ipsilateral internal mammary vessels from the first through the third intercostal spaces. The nodal PTV was generated by expanding the CTVs by 5 mm.

### Toxicity assessment and cosmetic results

Acute breast skin toxicity was assessed during each week of radiation treatment and at one month after radiation treatment. The maximum toxicity was graded according the common terminology criteria (CTC) for adverse events (version 3.0) based on the highest grade toxicity described in the weekly on-treatment notes, in the treatment summary or at the first follow-up visit after 1 month. All of these notes were reviewed for descriptions of patients-reported pain and for physical examination findings, including skin erythema, dry or moist desquamation and breast edema.

Cosmetic results were graded using 4-point scale according to the Harvard criteria based on patient self-assessments. Excellent or good outcomes reflected no identifiable or minimal radiation changes in the treated breast. Readily observable significant changes were scored as fair cosmetic. Severe radiation effects reflected a poor cosmetic score.

### Statistical analysis

For the collection of the dosimetric parameters, only patients with detailed records of acute skin reactions were included. Logistic regression analysis was used to test the predictors associated with Grade 2 or higher acute skin toxicity with the multibeam IMRT plan. The predictors included patient age (continuous), breast PTV, PTV_107%_, RLN irradiation, and chemotherapy.

Follow-up duration was calculated from the diagnosis of BC. Local recurrence (LR) was defined as any recurrence in the ipsilateral breast. Regional recurrence (RR) was defined as recurrence in the ipsilateral axilla, internal mammary, or supraclavicular node. Both LR and RR were defined as locoregional recurrence (LRR). Only the site of the first failure event was considered for the analysis of LRR. Recurrence events included LRR and distant metastasis (DM). Freedom from LRR and DM survival (LRRFS and DMFS), Recurrence free survival (RFS) and overall survival (OS) were all calculated using the Kaplan-Meier method. Multivariate Cox proportional hazard analysis with forward selection was used to study the prognostic factors of RFS. The prognostic factors were age, T stage and N status, grade, lymphovascular invasion and molecular subtypes. The molecular subtypes were defined as the luminal subtype (ER and/or PR positive), triple negative (TN) subtype (ER and PR negative, HER2 negative), and HER2 enriched subtype (ER and PR negative, HER2 positive). Metachronous contralateral BC was included in the analysis of secondary malignancy. Statistical analysis was performed using the SPSS software package (version 17.0), all *P* values were two-sided, *P* ≤ 0.05 was considered significant.

## CONCLUSIONS

To improve target conformity and reduce the volume of high-dose irradiation of normal tissues, a breast inverse IMRT technique with 4 to 7 fields was developed in our cancer center. Acceptable acute skin toxicity profile and cosmetic result was achieved. After 4 years of follow up, the LRRFS, DMFS, RFS and OS rates were excellent. Longer follow-up is needed.
